# GRKs and Epac1 Interaction in Cardiac Remodeling and Heart Failure

**DOI:** 10.3390/cells10010154

**Published:** 2021-01-14

**Authors:** Marion Laudette, Karina Formoso, Frank Lezoualc’h

**Affiliations:** INSERM UMR-1048, Institute of Metabolic and Cardiovascular Diseases, Université de Toulouse III—Paul Sabatier, 31432 Toulouse, France; laudettemarion@gmail.com (M.L.); karina.formoso@inserm.fr (K.F.)

**Keywords:** β-adrenergic receptors, Epac1, cAMP, G protein-coupled receptor kinases, signaling

## Abstract

β-adrenergic receptors (β-ARs) play a major role in the physiological regulation of cardiac function through signaling routes tightly controlled by G protein-coupled receptor kinases (GRKs). Although the acute stimulation of β-ARs and the subsequent production of cyclic AMP (cAMP) have beneficial effects on cardiac function, chronic stimulation of β-ARs as observed under sympathetic overdrive promotes the development of pathological cardiac remodeling and heart failure (HF), a leading cause of mortality worldwide. This is accompanied by an alteration in cAMP compartmentalization and the activation of the exchange protein directly activated by cAMP 1 (Epac1) signaling. Among downstream signals of β-ARs, compelling evidence indicates that GRK2, GRK5, and Epac1 represent attractive therapeutic targets for cardiac disease. Here, we summarize the pathophysiological roles of GRK2, GRK5, and Epac1 in the heart. We focus on their signalosome and describe how under pathological settings, these proteins can cross-talk and are part of scaffolded nodal signaling systems that contribute to a decreased cardiac function and HF development.

## 1. Introduction

G-protein-coupled receptors (GPCR) represent one of the largest superfamilies of transmembrane receptors and are involved in a wide variety of biological functions. Among the numerous GPCRs expressed in the cardiovascular system, β-adrenergic receptors (β-ARs) represent the most powerful system that regulates cardiac function and acutely increases the output of the heart [1]. In the classical paradigm, acute stimulation of cardiac β-AR by catecholamines (adrenaline, noradrenaline) induces the intracellular production of cyclic AMP (cAMP) and protein kinase A (PKA) activation, which plays a key role in the regulation of the contraction and relaxation of cardiac myocytes [2]. In addition to heterodimeric G protein activation and their downstream effectors, GPCRs promote receptor phosphorylation by G protein-coupled receptor kinases (GRKs) in an agonist dose-dependent fashion [3,4]. This process induces receptor desensitization through the binding of the scaffold protein β-arrestin (β-arr) to terminate receptor signaling.

Despite their capacity to confer sensitivity to acute adrenergic stimuli involved in physiological signaling, β-ARs also form a crucial part of the stress response pathways linked to and involved in cardiovascular disease, including heart failure (HF), a leading cause of death worldwide. Indeed, the excessive sympathetic nervous system activity observed in HF can promote the continued stimulation of cardiac β-ARs, which triggers adverse effects including over proportional increases in energy consumption, cell death, fibrosis, cardiomyocyte hypertrophy, and arrhythmia [5]. β-AR blockers are a mainstay in the therapy of HF, but the morbidity and mortality associated with HF still continue to rise. Therefore, it is important to elucidate the molecular players that couple the β-AR signaling route to pathological cardiac remodeling leading to HF, since novel findings might have therapeutic implications for novel treatment.

In addition to canonical receptor desensitization and downregulation, GRK2 and GRK5 are critical mediators of the molecular alterations that contribute to HF. The exchange protein directly activated by cAMP 1 (Epac1) is coupled to β-Ars, and various studies have revealed the involvement of this cAMP-binding protein in cardiac remodeling and HF [6,7,8,9,10]. In this review, after a brief description of cardiac β-ARs, we discuss the pathophysiological roles of GRK2, GRK5, and Epac1 in cardiac disease. We describe their signaling routes and interaction during cardiac stress conditions.

## 2. β-Adrenergic Regulation of Cardiac Function

### 2.1. G Protein-Coupled Receptors

G protein-coupled receptors (GPCRs) constitute the largest family of transmembrane proteins and mediate most cellular responses to hormones and neurotransmitters [11]. Structurally, GPCRs contain extracellular ligand-binding domains, seven membrane-spanning helices, and carboxy-terminal intracellular regions. They interact with membrane-associated heterodimeric G proteins that are composed of three subunits: Gα, which binds to nucleotides guanosine diphosphate (GDP) and guanosine triphosphate (GTP), Gβ, and Gγ subunits [1,12]. Following GPCR activation, both the Gα subunit bound to GTP and the Gβγ dimer dissociate from the receptor to activate downstream effector proteins, such as enzymes that produce second messengers and ion channels [1,12,13]. The catalytic Gα subunit will hydrolyze the bound GTP to GDP, leading to its reassociation with the Gβγ subunits and termination of the G-protein activation cycle [14].

Based on the sequence and functional similarities, Gα proteins are classified into four families: Gα_s_, Gα_i_, Gα_q_, and Gα_12/13_ [15]. GPCRs coupled to Gα_s_ and Gα_i_ regulate the activity of the membrane-bound adenylate cyclase (AC) that produces intracellular 3′-5′-cyclic adenosine monophosphate (cAMP) from adenosine triphosphate (ATP). The intracellular levels and diffusion of cAMP are further regulated by a large family of cyclic nucleotide-degrading enzymes named phosphodiesterases (PDEs) [16]. Cyclic AMP binds to protein kinase A (PKA) for the phosphorylation of target proteins or exchange proteins directly activated by cAMP (Epac), which function as guanine nucleotide exchange factors for the small GTPase Rap [17]. The coupling of GPCRs to Gα_q_ promotes phospholipase C (PLC) activation, which catalyzes the hydrolysis of phosphatidylinositol 4,5-bisphosphate (PIP2) to diacylglycerol (DAG) and inositol 1,4,5-trisphosphate (IP3). These second messengers are involved in a multitude of cellular processes, such as the mobilization of intracellular Ca^2+^ signaling from the endoplasmic reticulum via the IP3 receptor (IP3R) [18]. Finally, the GPCRs coupled to Gα_12/13_ are important regulators of cytoskeleton, cell junctions, and processes related to cell migration [12]. Like the Gα subunits, Gβγ dimers also mediate various cellular effects, and play key roles in the desensitization and recycling of GPCRs through the G protein-coupled receptor kinases (GRKs) and the scaffold proteins, β-arr [4].

### 2.2. Cardiac β-Adrenergic Receptors

Noradrenaline released from cardiac sympathetic nerve terminals and circulating adrenaline secreted by the adrenal glands regulate cardiac output through the stimulation of adrenergic receptors (ARs) [5]. These GPCRs are classified into two subtypes: α-ARs, which are mainly present in the vessels of the heart, and β-ARs, which are highly expressed in the myocardium. β-adrenergic receptors (β-ARs) provide the most powerful stimulation of cardiac function, and are involved in the pathophysiology of HF [5]. In the heart, β1-AR and β2-AR are the major β-AR subtypes, accounting for ≈80% and 20% of the total β-ARs, respectively [19,20]. The β3-AR is expressed at lower levels than β1-AR, and is coupled to nitric oxide synthase (eNOS and nNOS) and downstream cGMP/protein kinase G signaling to exert cardioprotective effects [21].

The excitation–contraction coupling is the process whereby an action potentially triggers cardiomyocyte contraction, followed by subsequent relaxation [2]. The acute stimulation of the β1-AR subtype increases the intracellular cAMP level to promote PKA activation, which phosphorylates key proteins of the excitation–contraction coupling such, as the L-type Ca^2+^ channel (LTCC), phospholamban (PLB), and the ryanodine receptor 2 (RyR2), to enhance cardiac contraction (inotropy) and relaxation (lusitropy) [2]. Specifically, PKA phosphorylates LTCC, which induces the entrance of external Ca^2+^ inside the cytosol of cardiomyocytes. The latter triggers a massive release of Ca^2+^ (through the opening of RyR2) from the sarcoplasmic reticulum (SR) to induce myofilament contraction. This process is known as Ca^2+^-induced Ca^2+^ release (CICR) [2,22,23]. Then, the PKA-dependent phosphorylation of PLB relieves its tonic inhibition of SR Ca^2+^ ATPase activity, thereby promoting Ca^2+^ uptake into the SR, and relaxation.

By increasing beat-to-beat Ca^2+^ transients via these mechanisms, the β1-AR mediates the sympathetic regulation of cardiac excitation–contraction coupling. The β2-AR activates Gα_s_ proteins, but also signals through Gα_i/o_ [24]. The inotropic positive effect of β2-AR in ventricles is moderate compared to β1-AR [25]. Indeed, although β2-AR signaling may increase Ca^2+^ influx via the L-type Ca^2+^ channel in some species, it does not affect calcium reuptake or overall cardiomyocyte contractility [18,26]. Consistently, catecholamines have no inotropic effect in β1-AR^−/−^ mice, unlike β2-AR^−/−^ mice [27]. Furthermore, in contrast to β1-AR, β2-AR activation may play a cardioprotective role in improving cardiac function and myocyte viability [28]. The compartmentalization of β1-AR and β2-AR signaling may explain these functional differences between the β-AR subtypes [29]. Indeed, the β2-AR is mainly concentrated in T tubules and lipid rafts, unlike β1-AR, which is more widely distributed across the plasmalemma of cardiomyocytes to induce a more diffuse cytosolic cAMP signal [29,30]. Recent findings suggest that the β2-AR compartmentalizes global β1-AR signaling into nanoscopic submembrane domains, converting β1-AR signaling from a global to a compartmentalized mode that prevents catecholamine from regulating intracellular Ca^2+^ transient and cardiac contraction [31]. It is suggested that β2-AR stimulation targets serine residues of the β1-AR C-terminus through GRK2, thereby facilitating PDEs recruitment and keeping cAMP in a submembrane nanodomain without reaching RyR2 [31].

As with all GPCRs, the β-AR signaling system adapts to prolonged stimulation by a process named desensitization, which leads to the rapid reduction of the receptor responsiveness within a few minutes. The phosphorylation by serine/threonine kinases, the GRKs of agonist-occupied β-AR, corresponds to the first step of the desensitization process. Then, GRKs enhance the affinity of the receptor for binding to the scaffold β-arr, which in turn inhibits the interaction of the β-AR with Gα_s_, thereby promoting the uncoupling of the β-AR. Beyond their classical function, compelling evidence indicates that GRKs and β-arr also play an important role in other processes, such as β-AR internalization, trafficking and resensitization, and contribute to the compartmentalization of cAMP signaling and its alteration in cardiac stress conditions (see paragraph 3 below [4,14,32,33]).

### 2.3. β-Adrenergic Receptor in HF

Although acute stimulation of the β-AR and the subsequent production of cAMP and PKA activation have beneficial effects on cardiac function, under elevated sympathetic drive, the chronic stimulation of β1-AR promotes the development of pathological cardiac remodeling. This process includes the modification of the morphology of the cardiac cavities associated with an increased size of cardiomyocyte, fibrosis, inflammation, and alterations of Ca^2+^ handling and energy metabolism [34]. In the long term, these changes alter cardiac contractility and can evolve into HF [35,36,37].

A major hallmark of the failing heart is an increase in β-AR desensitization, resulting in reduced adrenergic agonist effects on contractile performance [5,38,39]. In addition, the high levels of circulating catecholamines observed in HF are accompanied by a decrease in cardiac β1-AR density (around 50%) [40]. Although the expression of β2-ARs is not altered during HF, β2-AR is uncoupled from Gα_s_, being less efficient in producing cAMP. Increased myocardial Gα_i_ expression and activity have been reported in HF and contribute to altered β2-AR signaling and cardiac dysfunction [25,41]. β-AR signaling abnormalities in the failing myocardium also include altered cAMP compartmentation, as illustrated by the redistribution of the β2-AR from T tubules to the surfaces of cardiomyocytes, which generates a diffused cAMP gradient throughout the entire cytosol, similar to the β1-AR-cAMP signals [30,42]. Based on these data, it is suggested that β2-AR signaling may acquire the properties of the β1-AR response, and loses its cardioprotective effects, thus contributing to HF development [29,30].

β-ARs antagonists, also called β-blockers, reduce the mortality and morbidity in patients with chronic HF, and improve systolic function in the long term [35,43]. Although β-blockers have limited effectiveness in some HF patients and have adverse effects, they currently represent the first-line treatment for HF by improving heart function. At the molecular level, β-blockers therapy slows down β-AR desensitization and increases β-AR density in the setting of HF, as well as normalizing myocardial Ca^2+^ movements [4,44,45]. However, the exact mechanisms of the clinical benefit of β-blockers are not entirely clear, because it is not well understood whether they act by blocking or resensitizing the β-AR signaling system [24,35].

## 3. Pathophysiological Roles of GRK2 and GRK5 in the Heart

### 3.1. GRK Isoforms

The serine/threonine kinase GRK family is composed of seven members that are classified into three subfamilies based on sequence similarity and gene structure: GRK1 comprising GRK1 (rhodopsin kinase) and GRK7 (cone opsin kinase), the β-AR kinase (βARK) subfamily including GRK2 and GRK3, and the GRK4-like subfamily containing GRK4, GRK5, and GRK6 [46]. The GRK2, GRK3, and GRK5 isoforms are ubiquitously expressed, albeit to various extents in different tissues. GRK2 and GRK5 are the predominant isoforms expressed in the heart, whereas GRK3 and GRK6 members are present at low levels [47,48]. All GRKs are multidomain proteins containing three main regions: a central catalytic core, which is flanked by N- and C- terminal regions containing motifs involved in GRK regulation and key binding elements for effectors and protein–protein interactions [49]. The N-terminal part is well conserved between GRK members and is critical for the recognition of the activated receptor. The latter contains a regulator of the G protein signaling homology domain that plays a pivotal role in intracellular membrane anchoring. The C-terminal part differs between GRK subfamilies, and is involved in GRK subcellular membrane localization [50].

All GRKs are primarily localized to the cytosol and plasma membrane. They display receptor specificity in vivo [51]. The canonical role of GRKs is to phosphorylate activated GPCRs at their C-terminus to regulate their signaling via homologous desensitization. The resulting increase in the phosphorylation level of GPCR promotes the binding of β-arr to the activated receptor, which turns off and desensitizes the receptor [52]. Therefore, GRKs contribute, through this classical function, to the regulation and signal propagation of GPCRs [53]. However, compelling evidence indicates that GRKs can also act as signaling molecules by themselves and display a multitude of non-GPCR substrates and interactors, as well as regulatory functions in many signaling processes [3,54].

### 3.2. Role of GRK2 and GRK5 in Cardiac Remodeling and Heart Failure

GRK2 and GRK5, through their canonical and non-canonical actions, are important regulators of cardiovascular signaling and function in a normal heart. These predominant GRKs expressed in the myocardium are both shown to be upregulated in HF. Compelling evidence indicates that they contribute to the molecular alterations occurring during cardiac remodeling, and facilitate HF development [49]. Here below, we discuss the contribution of GRK2 to the progressive loss of the adrenergic and inotropic reserves of the heart during sympathetic overdrive, and the role of GRK5 in mediating cardiac hypertrophy during myocardial pressure overload.

#### 3.2.1. GRK2

GRK2 is mainly located in the cytosol, where it interacts with the free Gβγ subunits of activated heterotrimeric G proteins in order to translocate to the membrane and phosphorylate activated GPCRs [55]. Interestingly, GRK2 is also expressed in mitochondria, and this non-GPCR function of GRK2 regulates cardiomyocyte death and glucose oxidation [56,57].

At first glance, GRK2-increased activity may represent a cardioprotective mechanism against chronic adrenergic overdrive to terminate β-AR signaling. However, the observation that GRK2 expression and activity were augmented in human HF suggested that the loss of β-AR responsiveness observed in this disease could be due to GRK2-induced β-AR desensitization and uncoupling [1]. Similar upregulation of cardiac GRK2 was also reported in several experimental animal models of HF [58]. On the contrary, a recent report showed that transient downregulation of myocardial GRK2 occurred at early time points during ischemia–reperfusion injury and was detrimental to the heart [59]. These data suggest that there are complex dynamic changes in GRK2 expression levels during stress conditions which may influence the development of myocardial lesions. 

β-blockers therapy decreased GRK2 activity, suggesting that reduced GRK2 activity might be critical for β-blocker function in HF [60]. Of particular interest, the increase in GRK2 expression in lymphocytes can independently predict prognosis in patients with HF [61]. However, more direct evidence of the noxious role of increased GRK2 expression in the heart came from in vivo experiments using transgenic mice in contexts of both chronic hypertensive and ischemic disease [3,24,62]. A pioneer study demonstrated that transgenic mice overexpressing GRK2 at a similar level to that observed in HF displayed desensitized β-ARs and a cardiac inotropic reserve reduction [63]. Conversely, GRK2 genetic inhibition revealed the beneficial effects of GRK2 blockade against pathological cardiac remodeling induced by pressure overload or Angiotensin II infusion. A peptide inhibitor of GRK2-Gβγ interaction, commonly named βARKct or βARK1, has been largely used to investigate the functional impact of blocking GRK2 activity during cardiac stress conditions [4]. Overexpression of βARKct mitigated pathological cardiac remodeling and prevented HF development induced by myocardial infarction or pressure overload [58,63,64,65,66]. In addition, βARKct prevented the progression of HF and improved survival by reversing GRK2-mediated β-AR desensitization and internalization [65,66,67,68]. By analogy with βARKct, other molecules that disrupt GRK2–Gβγ binding to the membrane have been characterized. Among them, M119 a small molecule, and Gallein, a more chemically stable analog related to M119, block GRK2–Gβγ interactions and reduce GRK2 recruitment to the membrane of cardiomyocytes [69]. Both compounds can prevent the onset or the progression of HF and improve cardiac function in experimental models of HF such as chronic activation of β-ARs or myocardial pressure overload [70,71,72,73]. More recently, paroxetine, an antidepressant drug, was shown to block GRK2-mediated receptor phosphorylation and receptor desensitization. Administration of this serotonin uptake inhibitor in mice two weeks after myocardial infarction improved cardiac function and reduced adverse ischemic remodeling in mice [74]. Therefore, the aforementioned studies suggest GRK2 inhibition can enhance cardiac function and induce reversal of disease in various preclinical models of HF, indicating that targeting GRK2 represents a promising therapeutic approach for treating HF.

#### 3.2.2. GRK5

The role of GRK5 in cardiac β-AR signaling is less understood, although GRK5 can also desensitize these GPCRs, including β1-ARs [75]. In contrast to GRK2, GRK5 binds constitutively to the plasma membrane due to its phospholipid-binding domain and does not undergo agonist-dependent recruitment to the membrane. In fact, genetic approaches have shown the importance of GRK5 as a potential therapeutic target in cardiac hypertrophy leading to HF. Transgenic mice overexpressing GRK5 in the heart displayed β-AR desensitization [75]. Importantly, these mice had exaggerated cardiac hypertrophy and rapidly developed HF compared with control mice after myocardial pressure overload [76]. Conversely, global and cardiomyocyte-specific ablation of GRK5 significantly attenuated pathological cardiac hypertrophy and delayed the onset of HF induced by transverse aortic constriction [77]. Mechanistic analysis demonstrated that GRK5-induced cardiac hypertrophy was associated with its non-canonical activity in the nucleus of cardiomyocytes [76,77]. Indeed, GRK5 contains a functional nuclear targeting sequence and a nuclear export signal within its catalytic domain, allowing its nuclear translocation to regulate prohypertrophic gene expression [78,79]. 

**Figure 1:**
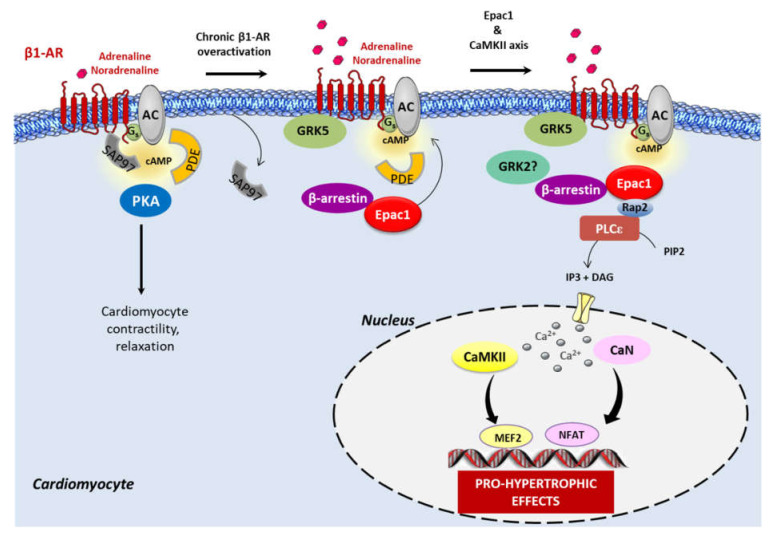
β1-AR signaling during chronic adrenergic stress. Acute stimulation of β1-AR induces cAMP production through the adenylyl cyclase (AC) and protein kinase (PKA) activation to regulate cardiomyocyte contraction and relaxation. Phosphodiesterases (PDE) regulate cAMP diffusion and concentration. In basal conditions, Epac1 constitutively interacts with β-arrestin in the cytosol of cardiomyocytes. Under the sustained activation of β1-AR, there is a switch of physiological cAMP-PKA signaling to cAMP-Epac1 pro-hypertrophic signaling. Mechanistically, the synapse-associated protein 97 (SAP97), a scaffold protein, is released from β1-AR and facilitates receptor signaling to the detrimental Epac1-Ca^2+^/calmodulin-dependent protein kinase II (CaMKII) axis. GRK5, and not GRK2, enhances agonist-induced dissociation of SAP97 from β1-AR. The complex β-arrestin–Epac1 is then recruited to the plasma membrane where Epac1 interacts with its effector, Rap2, which in turn stimulates the phospholipase C ε (PLCε). The subsequent production of 1,4,5-trisphosphate (IP3) and IP3-receptor stimulation induces Ca^2+^ elevation, which activates the Ca^2+^-sensitive proteins, CaMKII and calcineurin (CaN), and their downstream targets, the pro-hypertrophic transcription factors, myocyte enhancer factor 2 (MEF2), and the nuclear factor of activated T cells (NFAT). The role of GRK2 in Epac1 complex formation at the β1-AR has yet to be investigated.

It is suggested that following activation of Gα_q_ coupled receptor, such as α1-AR, the Ca^2+^ sensitive protein, calmodulin binds to the N-terminus of GRK5 and exposes the nuclear export signal of GRK5 to facilitate its nuclear export [80,81]. Once in the nucleus, GRK5 phosphorylates histone deacetylase-5 (HDAC5), leading to the de-repression of MEF2-induced hypertrophic gene transcription (Figure 1) [76]. Besides its HDAC kinase activity, GRK5 can directly bind to the DNA and act in a kinase-independent manner as a positive coregulator of NFAT-induced hypertrophic gene transcription under cardiac stress conditions (Figure 1) [79]. Of course, GRK5 might have additional unknown nuclear partners potentially involved in DNA binding and regulation of transcription. It is important to note that GRK5 acts as a pro-hypertrophic factor only under pathological conditions and does not influence the physiological growth of the heart even when highly overexpressed [82]. Therefore, GRK5 inhibition should not influence the development of physiological hypertrophy. Proof of concept of the detrimental effect of GRK5 activation in preclinical models of HF has open a novel avenue for the characterization of small-molecule inhibitors of GRK5 for the treatment of HF [4,50,83].

## 4. Role of Epac1 in Cardiac Pathophysiology

Cyclic AMP signals in the cell through different proteins that include PKA, hyperpolarization-activated cyclic nucleotide-gated (HCN) channels, Popeye domain-containing proteins (POPDCs), and the more recently discovered Epac proteins. The latter are cAMP-dependent guanine nucleotide exchange factors (GEFs), responsible for the exchange of GDP for GTP of the Ras-like small GTPases, Rap1, and Rap2 [84]. This family of protein is composed of Epac1 and Epac2, which are coded by two different genes, *RAPGEF3* and *RAPGEF4*, respectively. Epac proteins have differential patterns of expression during development, and display different and overlapping localizations in tissues. On one hand, Epac1 is a rather ubiquitous protein showing high expression levels in the heart and kidney. Meanwhile, Epac2 has a more restricted expression, and is found in the central nervous system and endocrine tissues [85].

In the cardiovascular system, Epac1 is expressed in various cell types, including cardiomyocytes, endothelial vascular cells, and cardiac fibroblasts [7]. The role of Epac1 in the heart has been largely studied in the last few years. Compelling evidence indicates that Epac1 is upregulated in cardiac stress conditions, and genetic and pharmacological studies have demonstrated that this cAMP-binding protein contributes to cardiac disease development [7,86].

### 4.1. Epac Structure and Activation

To date, only the crystallography structure of Epac2 has been achieved [87]. However, given the high structural sequence similarity between Epac isoforms, Epac1 modeling has been possible. Both Epac1 and Epac2 are multi-domain proteins with a core structure that is comprised of two lobes linked by a flexible region (PDB ID: 107F, 2BYV, 4F7Z, http://www.wwpdb.org/) [87]. The regulatory region located in the N-terminal part of Epac1 is composed of the disheveled-Egl10-pleckstrin (DEP) domain, which ensures Epac1 localization at the plasma membrane, and a cyclic nucleotide-binding domain (CNB-B), which is the most conserved region of the protein. CNB-B regulates Epac1 GEF activity. Epac1 lacks a second CNB domain (CNB-A) present in Epac2, which is involved in Epac2 localization [17,86]. This could explain the different subcellular localization of the two isoforms, Epac1 and Epac2. The catalytic domain located in the C-terminus presents a Ras association domain (RA), a Ras exchange motif (REM), and a cell division and cycle 25 homology domain (CDC25-HD). The latter is responsible for Epac1 catalytic activity and also contains s a nuclear targeting signal [7,17,88].

In the cell, Epac1 exists in different conformations. When the concentration of cAMP is low, Epac1 is inactive (Apo-EPAC1) and the regulatory region sterically hinders the CDC25 catalytic region [87]. Epac1 is also found in an intermediate inactive state that is more extended than the Apo conformation. This more relaxed conformation of Epac1 allows the interaction of cAMP to the CNB domain. Once cAMP is bound to the regulatory region, Epac1 changes its conformation to the active state where the regulatory region moves away from the catalytic region, thereby allowing the interaction of Epac1 with its direct effector Rap [87]. 

### 4.2. Epac Pharmacological Tools

The development and validation of pharmacological tools to modulate Epac1 activity has been at the center of discussion in the last years [86]. One of the challenges of this task is to achieve specificity of the compound avoiding off-target effects, especially on Epac2 and PKA that share structural similarities. The development of fluorescence-based assays, such as FRET (Fluorescence Resonance Energy Transfer) and BRET (Bioluminescence Resonance Energy Transfer) probes, assisted in the discovery of Epac1 pharmacological tools [89,90]. One important step towards Epac agonist characterization was the synthesis of the cAMP analog, 8-CPT, also called 007 (8-(4-chloro-phenylthio)-2’-O-methyladenosine-3’,5’-cAMP) [91]. 8-CPT and its acetoxymethyl ester derivative (8-CPT-AM) activate Epac1 with more efficiency than Epac2 or PKA [92]. The potency and the specificity shown towards Epac1 positions 8-CPT as the gold standard ligand for Epac1 activation in vitro and in vivo. However, other compounds are being developed; the first non-nucleotide agonist of Epac1, I942, for example, was isolated by Beck and collaborators (2019) [93]. Structural analysis showed that I942 was capable of interacting with the CNB domain of both Epac1 and Epac2, but it displayed partial agonist properties only towards Epac1 [93]. The group of Yarwood performed systematic modifications on I942 and then structure–activity relationship studies and found four new compounds (25e, 25f, 25n, and 25u) that showed better specificity and higher activity for Epac1 [94]. Another non-cyclic nucleotide molecule named SY009 was recently identified as a selective activator of Epac1 in cells [93]. Developing strong and specific activators of Epac1 will enable a more thorough study of its mechanism of action and function. 

It is worth mentioning that one major point of discussion in the field of Epac1 pharmacology is the uncertainty as to whether the focus should be placed on activating or inhibiting Epac1 for the treatment of different pathologies. Therefore, the search for Epac inhibitors, including Epac pan-probe or Epac isoform-specific antagonists, has been under intensive investigation [86]. The first Epac inhibitors were discovered ten years after the isolation of the Epac synthetic agonist, 8-CPT [91,95]. Among them, the small molecule named CE3F4 has become the Epac1 inhibitor of reference [86,95]. CE3F4 exists as a mix of (R)- and (S)-enantiomers, where (R)-CE3F4 is more potent than the (S)- enantiomer in inhibiting Epac1 activity [92,96]. This compound is highly specific to Epac1, and is able to prevent Epac1-dependent biological action in cultured cells. Concerning the heart, CE3F4 blocks many markers of cardiac hypertrophy and prevents the Ca^2+^ alteration associated with cardiac remodeling and arrhythmia in cultured cells, such as primary cardiomyocytes [86]. More recently, our laboratory developed another small molecule, AM-001, as a novel non-competitive inhibitor of Epac1 devoid of cytotoxicity and with high specificity towards Epac1 [6]. AM-001 interacts with an allosteric site formed by CDC25-HD and CNB and stabilizes an inactive state of Epac1, thus inhibiting its activity [86]. Importantly, the AM-001 compound protects the heart against acute and chronic models of cardiac stress (see chapter below, [6]).

### 4.3. Role of Epac in Cardiac Disease

One major step in uncovering Epac’s involvement in cardiac disorders was the development of Epac1 knock-out (Epac1^−/−^) mice. The cardiac function and morphology of Epac1^−/−^ mice were normal, and not different from those of the wild-type animal under resting conditions. However, the situation was different in stress conditions, since Epac1^−/−^ mice displayed better cardiac contractility, suggesting a detrimental effect of Epac1 in the heart [8,9]. In particular, Epac1^−/−^ mice were protected against cardiac hypertrophy and fibrosis induced by chronic activation of β-AR [8] and, interestingly, aging-related cardiac remodeling [9]. This protection was also confirmed upon injection of the Epac1 inhibitor, AM-001, that inhibited cardiac remodeling and improved cardiac function upon chronic β-AR stimulation in mice [6]. Consistent with these results, the direct or indirect activation of Epac1 with 8-CPT or β-AR stimulation induces augmentation in the cellular size and expression of prohypertrophic factors in primary cardiomyocytes [10,97]. With treatment with radiotherapy, the group of Vozenin and Morel found that the expression of Epac1 was increased, and that combined treatment with 8-CPT led to the early transcriptional activation of pro-hypertrophic genes (ANF and skeletal muscle α-actin) [98].

At the molecular level, Epac1 has been shown to form signalosomes in different subcellular compartments, including the nucleus and mitochondria, where it exerts differential functions (see below [17]). For instance, work from our laboratory found that Epac1^−/−^ mice were protected against cardiac ischemia–reperfusion injury [99]. This detrimental effect of Epac1, in this case, involves Epac1 localized in the mitochondria where it triggers apoptosis during ischemia [99]. Perinuclear or nuclear Epac1 induces epigenetic regulation and nuclear Ca^2+^ dysregulation that promote the development of hypertrophic gene program in cardiomyocytes [86,100,101,102]. Interestingly, Epac1 has also been related to the development of atrial and ventricular arrhythmias. SR Ca^2+^ leak, a major trigger of arrhythmias, and spontaneous SR Ca^2+^ release were attenuated in Epac1^−/−^ mice. Consistently, treatment of mice with the selective Epac1 inhibitor, CE3F4, significantly prevented atrial and ventricular arrhythmia [103]. Epac1 also contributes to cardiac rhythm disorders by increasing the expression of pro-arrhythmic channels (transient Receptor Potential Canonical (TRPC) 3/4 and potassium voltage-gated channel (KCN)) [104,105]. 

## 5. Epac1 and GRK Molecular Complex Formation in Cardiac Remodeling

As previously mentioned, chronic β-adrenergic activation results in desensitization and internalization of β-AR, which is accompanied by a decreased fight-or-flight response and detrimental changes, such as cardiomyocyte hypertrophy, apoptosis, and inflammation. Both GRKs and PKA induce the phosphorylation of agonist-activated β-AR in order to terminate signaling. This drives the recruitment of β-arr to the β-AR receptor, which sterically prevents further G-protein coupling to the receptor while promoting β-AR internalization [24,106]. Besides the desensitization and internalization of activated receptors, β-arr also acts as a nodal point, and further initiates the activation of many intracellular signaling routes independently of G protein activity through their function as scaffold proteins [14]. In this regard, β-arr in the heart may undergo GRK-arrestin signaling and play an important role in regulating normal and compromised cardiomyocyte function. Here below, we will describe how macromolecular complexes composed of β-arr, Epac1 and other signaling molecules regulate prohypertrophic signaling, and may be involved in the pathogenesis of HF.

### 5.1. Epac1, CaMKII and β-Arrestin Complex

The formation of a β-arr–Epac1 complex was initially reported in the heart [107]. Biochemical studies demonstrated that Epac1 constitutively interacts through its RA domain with the scaffold protein β-arrestin2 (β-arr2) in the cytosol of cardiomyocytes during basal conditions [107,108]. Although both β1-AR and β2-AR activate Epac1, only β1-AR stimulation allows the recruitment of Epac1-β-arr2 molecular complex to the plasma membrane, a process involving the activity of microtubules [109]. It is suggested that GRK-induced β1-AR phosphorylation resulting in β-arr recruitment might also stabilize Epac1 at the plasma membrane [107,110]. Consistent with studies showing that β-arr can facilitate binding to distinct signaling partners [106,111], the interaction of β-arr with the C-terminal tail of the β1-AR but not β2-AR induces a conformational change in β-arr that favors its interaction with Epac1 [107]. In close proximity to the β1-AR complex, Epac1 promotes the activation of the Ca^2+^ sensitive protein CaMKII, which promotes the phosphorylation of the histone deacetylase 4 (HDAC4). Phosphorylated HDAC4 is extruded out of the nucleus and relieves HDAC4 inhibition on the hypertrophic transcription factor, myocyte enhancer factor 2 (MEF2) [100,108]. 

**Figure 2:**
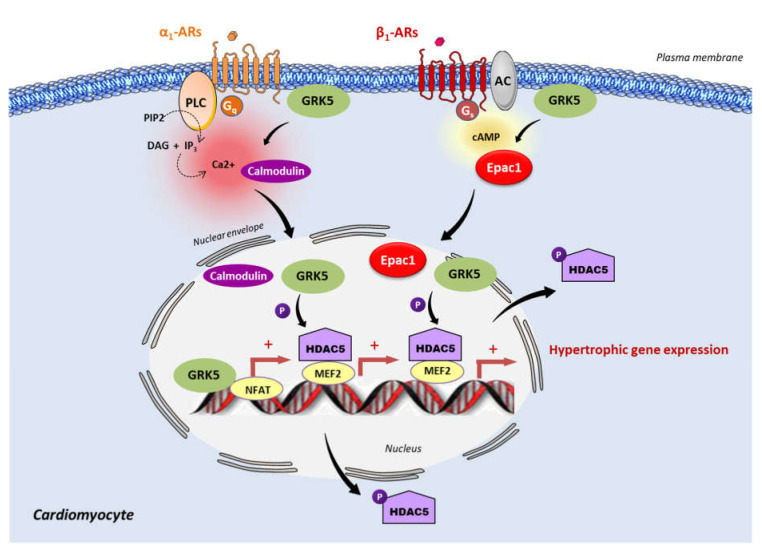
Epac1 and GRK5 non-canonical hypertrophic signaling. Activation of the Gα_q_ coupled receptor, α_1_-adrenergic receptor (α_1_-AR), promotes the intracellular elevation of Ca^2+^ through the phospholipase C (PLC) and the subsequent activation of calmodulin. GRK5 associates with Ca^2+^-calmodulin, leading to the translocation of GRK5 to the nucleus, wherein it promotes HDAC5 phosphorylation and subsequent myocyte enhancer factor 2 (MEF2) activation. During the chronic stimulation of β_1_-adrenergic receptor (β1-AR), Epac1 interacts with GRK5 and the Epac1-GRK5 molecular complex is exported to the nucleus of cardiomyocytes. There, GRK5 phosphorylates HDAC5, leading to its nuclear export, and thereby derepressing MEF2 transcriptional activity. GRK5 also acts as a coactivator of the nuclear factor of activated T cells (NFAT). MEF2 and NFAT are crucial transcription factors that promote cardiac hypertrophy.

The involvement of PKA in CaMKII activation seems to depend on the length and intensity of the stimulus. Indeed, a recent study using FRET-based biosensor showed that inhibition of PKA prevented acute stimulation of β1-AR-induced CaMKII activation [112]. However, CaMKII activity induced by prolonged activation of β-1AR was still maintained in the presence of PKA inhibitors [113]. Based on these observations, one could speculate that during chronic activation of cardiac β1-AR, there is a switch of β1-AR signaling from physiological cAMP-PKA activity to Epac1-CaMKII activity that promotes the development of cardiac remodeling and HF (Figure 2). Accordingly, expression and activity of CaMKII are increased during cardiac hypertrophy and HF [114,115]. In addition, chronic inhibition or gene deletion of CaMKII appears to have little effect on basal cardiac function or on acute responses to β-adrenergic stimulation but confer protection against pathological stresses known to be associated with chronic sympathetic activation [116]. 

Of particular importance, the interaction of Epac1 with β-arr2 is also regulated by the presence of the cAMP-specific phosphodiesterase 4 (PDE4) variant, PDE4D5. Indeed, Epac1 and PDE4D5 compete by steric hindrance for binding to β-arr2. Blocking the formation of the PDE4D5–β-arr molecular complex allows the translocation of the Epac1–β-arr complex to the activated β2-AR. Consequently, the β2-AR signaling switches to a β1-AR-like pro-hypertrophic signaling, and increases cardiomyocyte remodeling [108]. These data indicate that the differential interaction of Epac1 with β-arr2 contributes to the specificity of β1-AR and β2-AR signaling, and Epac1 compartmentalization participates in the distinct functions of the β-AR subtypes [17,108]. Interestingly, the interaction of β-arr seems not to be limited to Epac1, since a more recent study reported that the Epac2 isoform can directly interact with β-arr1 to regulate insulin secretion in pancreatic β cells [117].

As expected, Epac1 can undergo post-translational modifications, such as phosphorylation, that can regulate its activity. In this line, it has been demonstrated that in dorsal root ganglion neurons GRK2 controls Epac1-to-Rap1 signaling by phosphorylating Epac1 at Ser-108 in its DEP domain. This mechanism prevents the translocation of Epac1 to the plasma membrane in response to cAMP elevation, and underlies the protective effect of GRK2 on chronic inflammatory pain [118]. Whether such a non-canonical effect of GRK2 on Epac1 activity occurs in the context of cardiac remodeling has yet to be investigated.

### 5.2. Epac1 and GRK5

Since Epac1 and GRK5 are linked to hypertrophic signaling, recent studies have investigated their possible interaction in the regulation of this process. Recently, Laudette and collaborators (2019) showed that cardiac remodeling induced by chronic injection of the synthetic β-AR agonist, isoprenaline (ISO), increased GRK2 and GRK5 expression protein levels in mouse hearts [6]. Interestingly, the upregulation of GRK5, but not GRK2, was decreased in animals treated with AM-001, a specific pharmacological inhibitor of Epac1, suggesting that Epac1 specifically targets GRK5 [6]. At the molecular level, ISO increased Epac1–GRK5 interaction and promoted GRK5 nuclear import, while Epac1 inhibition with AM-001 prevented GRK5 nuclear translocation, to induce the nuclear accumulation of HDAC5. GRK5 acted as a downstream effector of Epac1 since the knock-down of GRK5 blocked the stimulating effect of Epac1 on prohypertrophic signaling. Following β-AR stimulation, Epac1 activation induces GRK5 nuclear import and HDAC5 nuclear export to promote prohypertrophic gene expression. Therefore, Epac1 seems to be required for non-canonical nuclear roles of GRK5 in maladaptive cardiac remodeling (Figure 1). Interestingly, it has been reported that GRK4 promotes cardiomyocyte apoptosis through the phosphorylation of HDAC4 during myocardial infarction [119]. Whether Epac1 functions with GRK4 to regulate cardiomyocyte death has yet to be investigated [119].

Adding complexity to the matter, a recent finding investigated the role of the synapse-associated protein 97 (SAP97) in β-AR signaling [112]. SAP97 is a multifunctional scaffold protein that binds to the C-terminal PDZ motif of β1-AR [120,121]. Xu and colleagues (2020) reported that the β1-AR-SAP97 molecular complex was reduced in HF. In addition, the authors demonstrated that cardiac-specific deletion of SAP97 yielded to spontaneous development of cardiomyopathy and exacerbated cardiac dysfunction induced by chronic β-AR stimulation and myocardial pressure overload in mice [112]. Mechanistic studies showed that loss of SAP97 as observed in HF promoted the recruitment of β-arr2 and CaMKII to β1-AR and switched on the β1-AR signaling to Epac-dependent CaMKII activity [112]. Yet, it was shown that GRK5 and not GRK2 enhanced ISO-induced dissociation of SAP97 from β1-AR, thereby facilitating the activation of the Epac–CaMKII axis and its detrimental functional and structural remodeling (Figure 2). At present, it is still unknown which Epac isoform is involved in this process but given the ascertained role of Epac1 in cardiac hypertrophy, one could imagine that the Epac1 isoform could be involved in this specific signaling. 

Another direction worth pursuing would be to examine the precise molecular mechanisms by which Epac mediates the activation of the Ca^2+^ sensitive protein, CaMKII, upon β1-AR stimulation. Does it depend on the activity of the downstream effector of Epac, the small GTPase Rap? Interestingly, a few years after the discovery of Epac, a study performed in HEK293 and neuroblastoma cells demonstrated that Epac activated the PLCε specifically through the Rap2 GTPase, resulting in the generation of inositol-1,4,5-trisphosphate (IP3) and the subsequent release of Ca^2+^ from intracellular stores [122]. Further studies showed that the Epac1–Rap axis could activate PLC, causing a Ca^2+^ increase via the IP3 receptor (IP3-R), to promote the activation of the Ca^2+^-sensitive dependent transcription factors involved in cardiac remodeling [100,123,124,125]. Alternatively, other downstream effectors of Epac1, including Rit and c-Jun NH2-terminal kinase (JNK), are activated by Rap1- and Rap2-independent mechanisms [126,127]. Whether these effectors are involved in the detrimental effect of Epac1 in cardiac remodeling has yet to be determined.

## 6. Conclusions

Evidence collected over the last decade indicates that GRK2, GRK5 and Epac1 contribute to the development and progression of HF, as illustrated here by experimental studies on pathological cardiac remodeling. A significant component of β-AR signaling is mediated through GRK and Epac1, and this pathway is particularly prominent under pathophysiological conditions. These promising results in terms of therapeutic innovation have stimulated the search for the identification of small molecules or peptides able to selectively inhibit the activity of a given GRK or Epac1 protein. In this line, several strategies have provided a proof of concept of the beneficial effect of blocking GRK2, GRK5 or Epac1 in animal models of HF. It is suggested that by normalizing β-AR signaling in human HF, GRK2-targeted therapeutic inhibition would restore the myocardial adrenergic reserve and improve cardiac function. Blocking the non-canonical action of GRK5 in the nucleus of cardiomyocytes prevents pathological hypertrophy. Finally, the pharmacological inhibition of Epac1 also ameliorates cardiac contractility, and attenuates cardiac remodeling and arrhythmia episodes by normalizing Ca^2+^ cycling. Whether GRK2, GRK5 or Epac1 inhibition would have a synergic effect on cardiac remodeling and HF has yet to be investigated. Therefore, further molecular studies are needed to characterize the GRK–Epac interactome and signalosome in subcellular compartments, so as to better understand how these proteins cross-talk to promote signaling alteration in HF.

## Data Availability

Not applicable.
